# Programming of the respiratory epithelium *in utero* - insight from the amniotic epithelial methylome

**DOI:** 10.1101/2025.10.02.25337047

**Published:** 2025-10-03

**Authors:** Patricia Agudelo-Romero, Thomas Iosifidis, James Lim, Nina Kresoje, David G. Hancock, Guicheng Zhang, Abhinav Sharma, Talya Conradie, Yuliya V. Karpievitch, Desiree T. Silva, Anthony Bosco, Susan L. Prescott, Peter N. LeSouëf, Elizabeth Kicic-Starcevich, Anthony Kicic, David J. Martino, Stephen M. Stick

**Affiliations:** 1 Wal-yan Respiratory Research Centre, The Kids Research Institute Australia, Perth, 6009, Western Australia, Australia.; 2 School of Molecular Sciences, The University of Western Australia, Perth, Western Australia 6009, Australia.; 3 European Virus Bioinformatics Centre, Friedrich-Schiller-Universitat Jena, 07737 Jena, Germany.; 4 School of Population Health, Curtin University, Perth, 6102, Western Australia, Australia.; 5 Centre for Cell Therapy and Regenerative Medicine, School of Medicine and Pharmacology, The University of Western Australia, Perth, 6009, Western Australia, Australia.; 6 Department of Respiratory and Sleep Medicine, Perth Children’s Hospital Perth, Perth, 6009, Western Australia, Australia.; 7 Medical School, The University of Western Australia, Perth, 6009, Western Australia, Australia.; 8 DSI-NRF Centre of Excellence for Biomedical Tuberculosis Research, SAMRC Centre for Tuberculosis Research, Division of Molecular Biology and Human Genetics, Faculty of Medicine and Health Sciences, Stellenbosch University, Cape Town, 7700, South Africa.; 9 School of Biomedical Sciences, The University of Western Australia, Perth, 6009, Western Australia, Australia.; 10 The Kids Research Institute Australia, Perth, 6009, Western Australia, Australia.; 11 School of Medical and Health Sciences, Edith Cowan University, Perth, 6027, Western Australia, Australia.; 12 Department of Paediatrics and Neonatology, Joondalup Health Campus, Perth, 6027, Western Australia, Australia.; 13 Asthma and Airway Disease Research Center University of Arizona, Tucson, 85721, Arizona, United States.; 14 Department of Immunobiology, The University of Arizona College of Medicine, Tucson, 85721, Arizona, United States.; 15 Nova Institute for Health, Baltimore, 21231, Maryland, United States.; 16 Department of Family and Community Medicine, University of Maryland, Baltimore, 21231, Maryland, United States.; 17 School of Paediatrics and Child Health, University of Western Australia, Perth, 6027, Western Australia, Australia.; 18 Occupation, Environment and Safety, School of Population Health, Curtin University, Perth, 6102, Western Australia, Australia.; 19 Department of Respiratory and Sleep Medicine, Perth Children’s Hospital, Perth, 6009, Western Australia, Australia.

**Keywords:** Epithelium, airway, amnion, in-utero, exposures, epigenetics, asthma, smoking, DNA methylation, EWAS, 1.12 Clinical asthma, 1.17 Epidemiology (Pediatric): Risk Factors < PEDIATRICS, 3.40 Airway epithelium < CELL AND MOLECULAR BIOLOGY, 3.05 Epigenetics < CELL AND MOLECULAR BIOLOGY, 3.02 Bioinformatics/Biological Computing < CELL AND MOLECULAR BIOLOGY

## Abstract

**Background:**

Dysregulation of the airway epithelium contributes to recurrent wheezing and asthma and may have developmental origins. Here, we investigated the relationship between the placental amniotic and nasal epithelial methylation landscapes to determine whether amniotic epithelium provides insight into fetal programming of respiratory tissue.

**Methods:**

We conducted high-throughput target-capture DNA methylation sequencing of 84 matched pairs of placental amniotic and neonatal nasal brushings samples within the Airway Epithelium Respiratory Illnesses and Allergy (AERIAL) cohort. Comparative analysis of tissue-specific methylation profiles, and conservation of methylation changes associated with gestational exposures (maternal smoking and maternal asthma), was explored.

**Results:**

Between amniotic and nasal tissues, we identified 4,897 differentially methylated regions (FDR ≤ 0.05 and log_2_FC ≥ |0.2|) that were generally hypermethylated in the nasal epithelium. Despite these extensive tissue-specific differences, filtering for loci with non-significant differential methylation (FDR ≥ 0.1) revealed 1,493,976 CpG loci (~20% of the measured methylome) with highly concordant methylation ratios levels between tissues (Pearson’s R ≥ 0.8). These loci included genes crucial to epithelial and lung development. Within these conserved regions, associations with maternal asthma and prenatal smoking were consistently represented in both tissues.

**Conclusions:**

The conserved methylome signatures support the use of amniotic tissue as a valuable tissue for investigating the developmental programming of airway vulnerability, potentially leading to early risk stratification and targeted interventions for childhood asthma.

## Introduction

Asthma is a chronic inflammatory airway disease, among the most frequent causes of hospital admissions in children and represents a significant global disease burden (1–3). A substantial body of evidence suggests prenatal exposures play a critical role in shaping newborn lung function (4), and contribute to the airway epithelial cell dysfunction observed in children with asthma (5). Intrauterine exposure to air pollution and tobacco smoke are well-established risk factors for childhood asthma that impair fetal lung development and increase airway hyperresponsiveness in offspring (6). Similarly, poorly controlled maternal asthma in pregnancy has been associated with childhood wheeze and increased risk for asthma in offspring through alterations in fetal development (7) Animal models also suggest intrauterine chronic inflammatory conditions might directly influence fetal-placental development (8) and aberrant prenatal programing of the airway epithelium tissues (9). Understanding these developmental programming mechanisms is a research priority needed to facilitate early detection and targeted interventions.

Epigenetic factors such as DNA methylation play a crucial role in fetal lung development, and changes in DNA methylation have been observed in the airway epithelium in children with asthma (10, 11). Environmental exposures such as cigarette smoke and pollution induce reversible epigenetic changes in airway epithelial cells via oxidative stress (12, 13), disrupting developmental gene expression patterns, and contributing to asthma development (14, 15). Methylation may therefore be an important epigenetic mechanism through which to understand the complex interplay between the prenatal environment and fetal epithelial programming in the development of childhood asthma.

A key challenge to studying the prenatal origins of epithelial dysfunction relates to the challenges of sampling the newborn airway epithelial cells, which are difficult to obtain before the onset of key postnatal events such as colonization of the mucosal surfaces, and exposure to airborne antigens. To address this, we explored the utility of fetal amniotic epithelial tissue as a surrogate for airway epithelial cells (16). The placental amnion is exposed to the maternal environment and may harbor epigenetic signatures (17, 18) that reflect processes occurring in the developing fetal respiratory tract. Amniotic epithelium cells possess stem cell like characteristics and are also attractive targets for cellular therapy (19). In this observational study, we explored the landscape of DNA methylation in amniotic epithelium and matched nasal epithelial samples from a cohort of healthy term newborns from the Airway Epithelium Respiratory Illnesses and Allergy (AERIAL) study (20). We conducted a comparative analysis of genome-wide DNA methylation profiles, and examined the relative conservation of DNA methylation patterning at specific genes crucial for lung and epithelial development. We also explored whether methylation changes associations with key maternal exposures that influence asthma development were conserved in both tissues. Our results inform ongoing developmental programming studies on epithelium vulnerability.

## Methods

### Study Design

AERIAL (20) is a prospective birth cohort nested within The ORIGINS birth cohort (21, 22). Matched pairs of amniotic membrane biopsies, nasal brushings and maternal urine were obtained from 84 newborns and their mothers. Maternal asthma history and tobacco smoke exposure during pregnancy was ascertained from at least 2 consecutive questionnaires administered at 20, 28 and 36 weeks of pregnancy. This study was conducted in accordance with the Declaration of Helsinki and was approved by the Ramsey Health Care HREC WA-SA (#1908). All parents, guardians, or next of kin provided written informed consent to participate in this study and collect maternal and newborn samples for the downstream analyses presented in this article.

### Sample Collection and Processing

Placentas were processed within 48 hours post-birth on average (20.4 ± 13.1 hours [SD (Standard Deviation)]), with the chorion membrane manually separated (23) and the amnion membrane sampled. Matched nasal epithelial samples from newborns were collected within six weeks from birth (15.9 ± 7.9 days [SD]) post-birth as detailed in the study protocol (20). All samples were cryopreserved at −80°C until DNA extraction was performed.

### Nucleic Acid Isolation

Genomic DNA was extracted from both nasal brushings and amniotic membrane samples using the chemagic 360 automated nucleic acid isolation system (Revvity, Baesweiler, Germany) and the chemagic^™^ DNA Blood 400 Kit H96 (Revvity, part# CMG-1091), following the manufacturer’s instructions, and then stored at −80 °C until analysis. DNA quantity was assessed using the Qubit HS dsDNA Assay (Q32854, Thermo Fisher Scientific) on a Qubit fluorometer (Thermo Fisher Scientific, Waltham, MA) (Figure E1 in the online supplement).

### DNA methylation measures

Libraries were constructed using capture DNA methylation sequencing with enzymatic conversion (EM-seq) and target enrichment employing the TWIST Human Methylome Panel. Libraries were prepared from 200 ng of genomic DNA using the NEB Next Enzymatic Methylseq Library Preparation Protocol (Twist Bioscience and New England Biolabs, CA, USA) following the manufacturer’s instructions. Targeted capture was performed using the Twist Targeted Methylation Sequencing protocol. Pre-capture libraries were pooled in 8-plex format and hybridized at 60°C for 16 hours. Subsequently, the hybridized pools were washed, and PCR amplified for six cycles according to the manufacturer’s protocol. Finally, capture libraries were sequenced at the Genomics WA facility on an Illumina NovaSeq 6000 (Illumina, CA, USA) using a pair-end configuration with 150 base pair (bp) length reads (Figure E1 in the online supplement).

### Bioinformatic Analysis

Bioinformatics analyses utilized computing and data resource provided by the Australian BioCommons Leadership Share (ABLeS) program (24) and Pawsey Supercomputing Research Centre (25, 26). Figure E1 summarizes the main steps carried out during the bioinformatics analyses. Briefly, raw methylation FASTQ files were processed using the nf-core/methylseq v2.3.0 pipeline. Methylseq pipeline was executed under Nextflow v23.04.2 (Di Tommaso et al., 2017) and the Human Genome Reference Consortium Human Build 38 (GRCh38) using the BWA-meth/MethylDackel workflow. Subsequent metrics and filtering were performed using target-methylseq-qc pipeline v2.1.0 (28), which included targeted methylation coverage assessment via *Picard-Profiler* mode and generation of targeted BED files based on BED intervals from the TWIST Human Methylome Panel using *bed-filter* mode. The Kids Research Institute Australia cluster and Google Cloud infrastructure were utilized for further analyses. Relevant scripts for data pre-processing are available on GitHub (https://github.com/wal-yan/AERIAL/tree/main/RespEpithelium_Methylome).

### Statistical Analyses

Statistical analyses were conducted in R language v4.3.2 and RStudio v2023.03.0+386 (29). Figure E1 in the online supplement illustrates the flow of the analyses performed. Where specific packages are not mentioned, analyses were conducted using base R functions. During data quality control (QC), 2.58% of CpG sites (n=204,963) with zero coverage in any sample were removed. Additionally, 9.70% of CpG loci (n=770,067) with very low coverage (<5) or extremely high coverage (>500) were excluded. The remaining 87.72% of CpG sites (n=6,961,516) that passed QC were used for subsequent downstream analysis. Mitochondrial and non-standard chromosomes were removed from the dataset. Methylation ratios were derived from sequencing counts and expressed as beta (β) as follows:

$$\frac{methylated\;alleles}{\left(unmethylated\;+\;methylated\right)\;*\;100}$$

With log_2_ transformation to *M-*values for statistical analysis. Sample quality control was performed by sex inference by extracting methylation calls on sex chromosomes and comparing to self-reported sex to identify potential sample mix-ups. Dimensional reduction was conducted using Principal Component Analysis (PCA) on M values (Figure E1, Step1, in the online supplement). Differential methylation was evaluated both within regions (differentially methylated regions, DMRs) and at individual CpG sites (differentially methylated positions, DMPs) using linear regression. DMRs were detected using the DMRcate package v2.16.1 (30) and the model built using tissue type as the primary variable and adjusting only for gender, as DMRcate method corrects per sample using modelMatrixMeth function from edgeR package (31), therefore participant ID were not included to avoid overfitting. To define DMRs, a minimum of 50 CpG sites was used, applying a stringent threshold for genome-wide significance (Fold Discovery Rate (FDR) ≤ 0.01 and mean difference ≥ |1|) (Figure E1, Step2, in the online supplement). For CpG-level analysis, the limma package v3.58.1 (32) was used to construct a linear model with tissue type as the primary variable including gender as fixed effect. We also adjusted for the non-independence of samples from the same individual by applying the *duplicateCorrelation()* method from the limma, using individual ID as a blocking variable. The estimated correlation was included during model fitting using *lmfit()* to improve the accuracy of differential analysis. Tissue-specific DMPs were identified using a threshold of FDR ≤ 0.05 and log_2_ Fold Change (log_2_FC) > |0.2| (Figure E1, Step3, in the online supplement). To identify conserved CpGs, we first retained loci with no significant differences between tissues (FDR ≥ 0.1). Among these, we then selected CpGs with high concordance in methylation levels between tissues (Pearson’s correlation, R ≥ 0.8). Note that the FDR threshold refers to the differential methylation analysis between tissues, not the correlation with exposures. Gestational exposures (maternal smoking and maternal asthma) were then evaluated separately within conserved regions using the same limma-based linear modelling approach describe above, adjusting for sex and accounting for repeated measures by participant ID. Significant associations were defined as FDR ≤ 0.05 and log_2_FC ≥ |0.2| (Figure E1, Step 4, in the online supplement).

DMPs were annotated to genes using the annotatr v1.28.0 (33), or internal functions for DMR calling using DMRcate v2.16.1 (30). Gene Ontology (GO) pathway enrichment analysis was conducted using the rGREAT v2.4.0 (34), applying the ‘Two nearest genes’ rule where gene regulatory domains were defined by extending in both directions to the nearest transcriptional start site, but no more than 20kb in either direction. Statistically enriched pathway terms were summarized using the Simplify Enrichment package v1.12.0 (35). Supervised Partial-Least Square (PLS) Discriminant Analysis (DA) was implemented with mixOmics v6.26.0 (36) to assess associations with maternal asthma history and *in utero s*moking exposure. Visualization of BAM files was performed using the Integrative Genome Viewer (IGV) software v2.8.9 (37)

### Urinary Cotinine Levels

Maternal smoking status was determined via positive response to questionnaire and validated with urinary cotinine levels (38) . Urinary cotinine levels were measured using the Salimetrics^®^ High Sensitivity Salivary Cotinine Enzyme Immunoassay Kit (Cat No. 1–2002-5, SALIMETRICS, State College, Pennsylvania 16803, USA), following the manufacturer’s protocol. Urine samples collected at 20 and 36 weeks of pregnancy as part of the ORIGINS biobank protocol (21, 22) were preserved at −80°C until analyzed. A standard curve was then established using a 4-parameter non-linear regression curve fit, and cotinine concentrations calculated using GraphPad Prism software v9.3.1. The assay lower limit of cotinine detection was 0.15 ng/mL. Positive urinary cotinine tests were >10 ng/mL with those below 10 ng/ml as non-smokers.

### Data Deposition

Due to ethical and privacy restrictions, raw sequencing datasets cannot be made available. However, de-identified methylation beta values sufficient to reproduce the findings are available upon request from the authors, while associated scripts are publicly accessible on GitHub (https://github.com/wal-yan/AERIAL/tree/main/RespEpithelium_Methylome).

## Results

### Demographics and Sequencing quality

The characteristics of the study population are presented in Table 1. While the newborns’ ethnic backgrounds were diverse, the cohort was predominantly Caucasian, with 51.2% of mothers and 48.8% of fathers identifying as such. Newborns had a mean gestational age of 39.12 ± 1.28 weeks [SD], with three preterm births recorded in the cohort, occurring between 35 and 36 weeks of gestation. Sequencing quality statistics were similar across tissues (Table 2), with low duplication rates and 93% of bases covered at a depth of at least 30X. The average number of paired-end sequences generated was approximately 183 ± 28.90 [SD] million for amnion samples and 168.67 ± 33.50 [SD] million for nasal samples. The methylation status was assessed across 7,936,546 million individual CpG dinucleotides, with 6,961,516 remaining after quality control filtering. Fold-80 base penalty metrics were between 1.2–1.6 across all samples indicating good uniformity of coverage for low-diversity libraries.

### Differential Methylation Landscape Between Amniotic and Nasal Tissues

**Figure 1.**
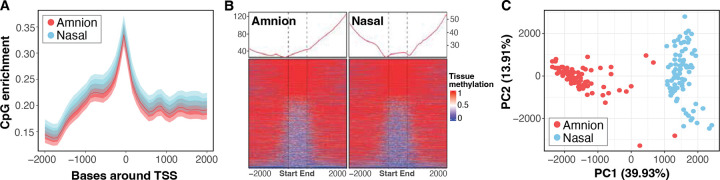
Comparative analysis of DNA methylation landscapes in amnion and nasal tissues. (A) Density plot illustrating CpG enrichment around Transcriptional Start Sites (TSSs). (B) Methylation levels of CpG islands with ± 2 kb. CpG islands are rescaled to the same width from Start to End vertical lines. (C) Principal component analysis (PCA) of genome-wide DNA methylation tissue profiles reveals distinct clustering of Amnion (red) and Nasal (blue) samples. Principal Component 1 (PC1) accounts for 39.93% of the variance.

We began by examining the general features of epithelium methylation landscapes between amniotic and nasal tissues, which revealed similar enrichment of methylated CpG sites around transcriptional start sites (Figure 1A), and the distribution of methylated and unmethylated CpG islands was similar across tissues (Figure 1B). These patterns were consistent across gene promoters and other regulatory features such as gene enhancers (Figure 1A and Figure E2A in the online supplement). Despite the overall similarities in CpG enrichment, dimensional reduction analysis of all 6,961,516 CpG sites across the genome revealed tissue type as the primary source of variation in DNA methylation profiles (PERMANOVA: pseudo-F=91.52, R^2^ 0.36, p-value=0.001) suggesting substantial tissue-specific differences (Figure 1C).

**Figure 2.**
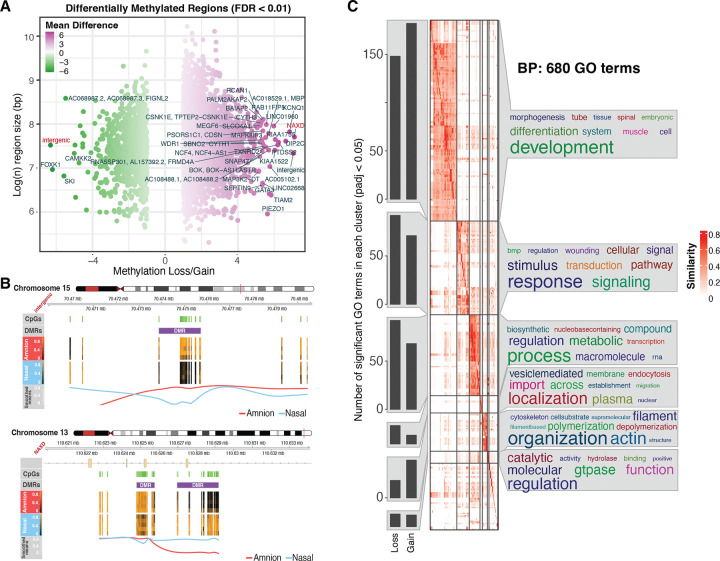
Regions differentially methylated between amniotic and nasal samples are enriched in cell development pathways. (A) Volcano plot displaying differentially methylated regions (DMRs) between amnion and nasal tissues. The x-axis represents the mean methylation difference, and the y-axis represents the log_2_-transformed region size. Genes associated with the most significant DMRs are labelled in red. (B) Genomic tracks showing methylation loss or gain in specific regions of Chromosomes 13 (*NAXD*) and 15 (Intergenic region), respectively. CpG islands are marked in green. The line graph summarizes the methylation difference between amnion (red) and nasal (blue) tissues across the region. (C) Gene Ontology (GO) enrichment was calculated using genes near DMRs between amniotic and nasal tissues, followed by a similarity matrix analysis. The similarity heatmap clusters significant GO terms into related Biological Process (BP), with colour intensity representing the significance of enrichment. Similarity of related terms within the same cluster is shown in the heatmap.

To determine the extent of tissue-specific differences in DNA methylation patterns, we conducted differential analysis comparing amniotic and nasal epithelium at individual CpG sites and identifying the differentially methylated regions (DMR). This analysis revealed 716,075 individual CpGs that contributed to 4,897 genome-wide significant DMRs (Table E1 in the online supplement). Notably, in the nasal epithelium most regions exhibited higher methylation, at 3,847 regions (78.50%), while 1,050 regions (21.50%) showed a loss in methylation, compared to the amniotic epithelium. The volcano plot highlights the most hypomethylated region in amnion relative to nasal tissue as an intergenic region on chromosome 15 (chr15:70473876–70475721), while the most hypermethylated region was within the *NAXD* gene on chromosome 13 (chr13:110626965–110629183) (Figure 2A). Genomic track visualization revealed broad and consistent methylation differences across these loci (Figure 2B). The intergenic region on chromosome 15 exhibited higher methylation levels in amnion, whereas *NAXD* on chromosome 13 showed increased methylation in nasal tissue (Figure 2B). To gain broader insights into the biological processes associated with these methylation differences, we annotated DMRs to the nearest genes and performed Gene Ontology (GO) enrichment analysis. This revealed strong enrichment of pathways related to development and differentiation, with additional ontologies related to stimuli and metabolic processes (Figure 2C and Figure E2B in the online supplement).

### Conservation of Methylation Across A Subset of Lung Specific Genes

**Figure 3.**
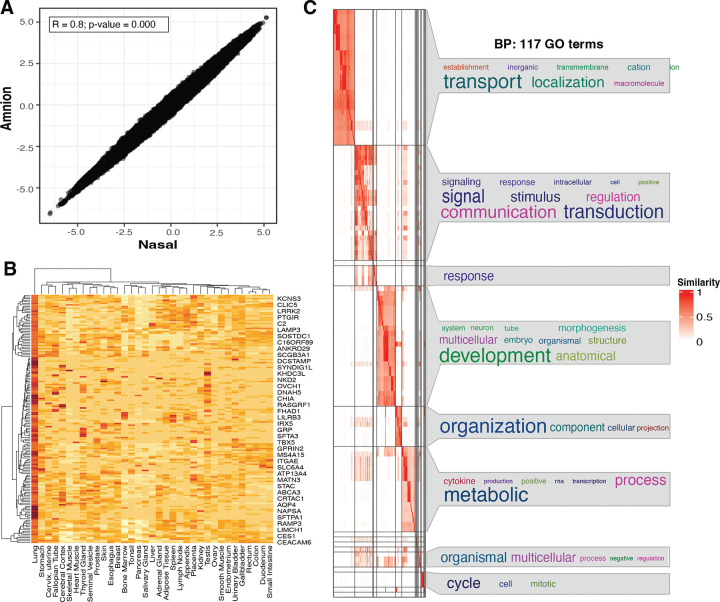
Conserved methylation landscapes were enriched in cell differentiation and morphogenesis pathways. (A) Scatter plot comparing DNA methylation levels between amnion and nasal tissues. Each dot represents a genomic region, with its position indicating the methylation level in each tissue. The strong positive correlation (Pearson’s R ≥ 0.8, p-value = 0.000) suggests a high degree of conservation in methylation patterns between these tissues. (B) Heatmap illustrating the expression levels of lung- and epithelial-specific genes across various tissues. Each row represents a gene, and each column represents a tissue. The colour intensity indicates the level of expression, with red representing high expression and blue representing low expression. Data are derived from the GTEx catalogue. (C) Gene Ontology (GO) enrichment was calculated using genes near CpG sites from the conserved methylation signature, followed by a similarity matrix analysis. The similarity heatmap displays GO terms associated with Biological Process (BP), with colour intensity representing the significance of enrichment.

We identified a conserved methylation signature (Figure 3A and Table E2 in the online supplement), comprising 1,493,976 CpG loci, representing approximately 21% of the total CpG landscape (n=6,961,516). This conserved pattern was distributed across all genomic regulatory features, with the highest representation in introns (20.09%), CpG islands (14.10%), and promoters (12.10%) (Figure E2A in the online supplement). To explore whether there are conserved methylation patterns in genes biologically relevant to epithelial and lung development, we queried the Genotype-Tissue Expression (GTEx) consortium database (https://gtexportal.org/home/) to extract a set of genes uniquely expressed in lung tissue compared to all other anatomical sites (total 459 genes). Among these gene sets, 159 genes (~34.6%) exhibited highly concordant methylation patterns (Pearson’s R ≥ 0.8) between the two tissues and general conservation of the methylation landscape (Figure 3B). For instance, Surfactant Protein A1 (*SFTPA1)* exhibited intermediate levels of DNA methylation in both amnion and nasal tissues (Figure E2B in the online supplement), while the epithelial specific gene, Secretoglobin Family 3A Member 1 (*SCGB3A1),* known to regulate cell proliferation was predominantly hypomethylated in both tissues (Figure E2B in the online supplement), indicating conservation in subsets of biologically relevant genes. We conducted a pathway enrichment analysis of the conserved signature reveling enrichment of terms related to anatomical and morphogenic processes, cellular organization, metabolism and cell cycling, as well as the regulation of signal transduction and stimulus response (Figure 3D and Figure E2C in the online supplement).

### Amnion tissue preserves methylation signatures associated with exposures during pregnancy

**Figure 4.**
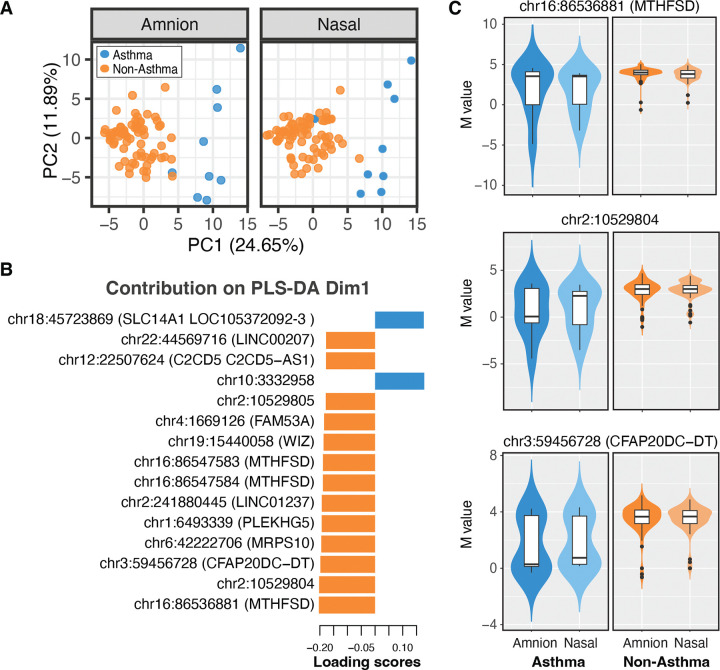
Methylation changes associated with maternal asthma in the conserved signature. (A) Principal Component Analysis (PCA) of DNA methylation variation in newborn amnion and nasal tissues, showing the first two principal components (PC1 and PC2). Samples are coloured based on maternal asthma history, with asthma in orange and non-asthma in blue. (B) Bar plot of loading scores from Dimension 1 (Dim1) of a Partial Least Squares Discriminant Analysis (PLS-DA), highlighting the top 15 differentially methylated CpG sites associated with maternal asthma. (C) Violin plots of the top three CpG sites identified by PLS-DA, showing the distribution of methylation levels (expressed as M-values) at features associated with maternal asthma in newborn amnion and nasal tissues, further stratified by maternal asthma history during pregnancy status.

Within the conserved portion of the methylome (Table E2 in the online supplement), we investigated statistical associations with two well-characterized exposures known to increase the risk of asthma development in offspring: maternal asthma history and *in utero s*moking exposure (39). In this cohort 9 out of 84 mothers reported a history of asthma. Binary logistic regression analysis, adjusted for multiple comparisons (FDR ≤ 0.05), identified a small but significant associations at 31 CpG sites (25 hypermethylated and 6 hypomethylated) with maternal asthma (Table 3 and Table E3 in the online supplement). Principal component analysis (PCA) of the conserved CpG sites significantly associates with maternal asthma history showed that this exposure was the major driver of variation in DNA methylation at these CpGs in both tissues (PERMANOVA: pseudo-F=29.09, R^2^ 0.15, p-value=0.001) (Figure 4A). Enrichment analysis linked these DMPs to pathways involved in cell communication, signal transduction, and response to stimuli. We applied Partial Least Squares - Discriminant Analysis (PLS-DA), which identified the 15 most relevant features contributing to group separation (Figure 4B). Notably, methylation levels of 13 CpGs decreased in the maternal asthma group, while two CpGs increased (Figure 4C). We visualized the top three significant CpG sites: chr16:86536881 (*MTHFSD*), chr2:10529804, and chr3:59456728 (*CFAP20DC-DT*), exhibiting the largest effect sizes which demonstrated consistency across both amnion and nasal tissues in relation to maternal asthma history (Figure 4C).

**Figure 5.**
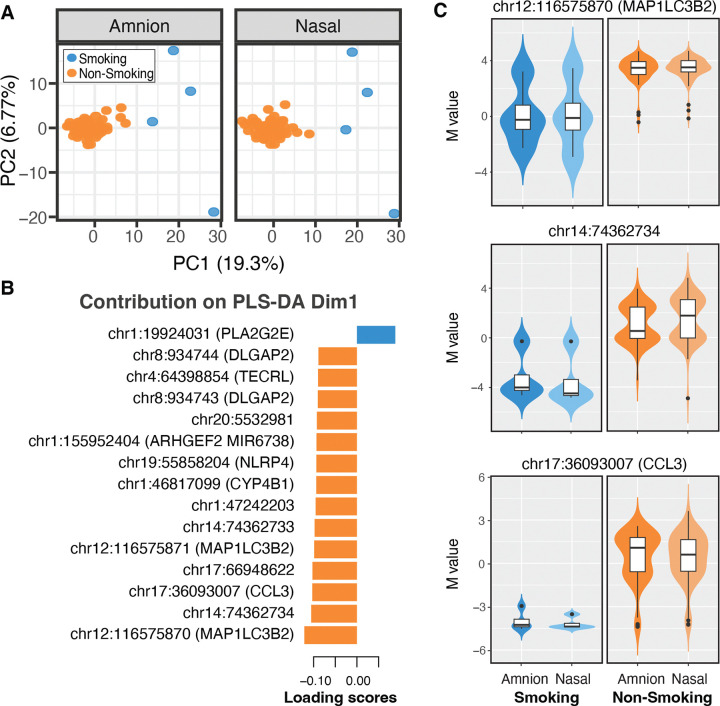
Methylation changes associated with maternal smoking exposure in the conserved signature. (A) Principal Component Analysis (PCA) of DNA methylation variation in newborn amnion and nasal tissues, showing the first two principal components (PC1 and PC2). Samples are coloured based on maternal smoking exposure, with smoking exposure in orange and non-smoking exposure in blue. (B) Bar plot of loading scores from Dimension 1 (Dim1) of a Partial Least Squares Discriminant Analysis (PLS-DA), highlighting the top 15 differentially methylated CpG sites associated with maternal smoking exposure. (C) Violin plots of the top three CpG sites identified by PLS-DA, showing the distribution of methylation levels (expressed as M-values) at features associated with maternal smoking exposure in newborn amnion and nasal tissues, further stratified by prenatal smoking status.

Next, we used the urinary cotinine measurements at 20 and 36 weeks of gestation as a marker of intrauterine smoke exposure. In our cohort, 4 of 88 mothers had cotidine levels consistent with tobacco smoking. Binary logistic regression analysis identified 164 CpG significant associations (126 hypermethylated and 38 hypomethylated) with maternal smoking exposure (Table 4 and Table E4 in the online supplement). Principal component analysis (PCA) of the conserved CpG sites significantly associated with smoking exposure identified it as primary source of the variation in DNA methylation in both tissues (PERMANOVA: pseudo-F=27.23, R^2^ 0.15, p-value=0.001) (Figure 5A), with significant enrichment of pathways involved in regulation of localization, cell activation, proliferation, and lung development. By applying PLS-DA the 15 most relevant features contributing to group separation (Figure 5B) comprised 14 CpG sites with decreased levels in the exposed group, while one CpG increased. Visualization of the top three significant CpG sites: chr12:116575870 (*MAP1LC3B2*), chr14:74362734, and chr17:36093007 (*CCL3*), also demonstrated the consistency of these changes across both amnion and nasal tissues between individuals with and without prenatal smoking exposure (Figure 5C).

## Discussion

The goal of this study was to explore the utility of placental amnion tissue as an easily accessible fetal-derived tissue for studying fetal programming of epithelial function in the context of asthma development. To achieve this, we investigated the relationship between the placental amniotic and nasal epithelial methylation landscapes in *ex vivo* samples from newborns. The key observations from this study are as follows.

Substantial tissue-specific methylation differences existed between the two tissues, characterized by a predominantly hypomethylated profile (reduced methylation) in amniotic epithelium samples. Given that DNA methylation levels typically increase in more terminally differentiated cell types, reflecting a more restrictive chromatin structure, this hypomethylation in amniotic tissue aligns with its more pluripotent state and stem-cell like characteristics reported in regenerative medicine studies (19). Despite these considerable differences, general features of the methylation landscape were similar, including enrichment of methylated CpGs around transcriptional start sites and low methylation across CpG Islands. consistent with patterns reported across multiple human somatic tissues (40). At the same time, histone modifications have been reported to contribute to epithelial plasticity, which is involved in both development and disease progression (41, 42). This divergence in epigenetic control may facilitate tissue-specific adaptations and responses to environmental changes, underscoring the dynamic nature of the epigenome.

Around 20% of the regions captured by the EM-seq target capture assay exhibited very high concordance of methylation levels, which we deemed the ‘conserved methylome’ between amniotic and nasal tissues (Figure 3A, Table E2 in the online supplement). This shared methylation signature may reflect common developmental and regulatory pathways shaped by the intrauterine environment. Amniotic fluid contains bioactive molecules that play essential roles in fetal development, particularly influencing critical cellular processes in lung epithelial cells (43). Additionally, the amniotic membrane contributes to the production of surfactant proteins and lipids vital for fetal lung maturation (44). Notably, within this conserved fraction, genes that play a critical role in lung development were represented, supporting the utility for amniotic tissue as a window for developmental programming of the respiratory epithelium (Figure 3B). However, we acknowledge that the full spectrum of biologically relevant genes with conserved methylation patters remains to be determined, a limitation of the present analysis. Future studies using more comprehensive and unbiased methods, such as whole-genome bisulfite sequencing, could help uncover additional loci of interest.

Moving beyond this comparative analysis, we examined how maternal exposures known to influence the development of asthma in early life are reflected in the conserved methylation patterns of both tissues (Figure 5 and Figure 6). Strengthening support for the utility of amniotic tissue to reveal intrauterine influences on respiratory development, we report that statistical associations were equivalent across tissues within this conserved fraction of the methylome (Table E3 in the online supplement). While previous studies have reported epigenetic alterations in the offspring linked to maternal asthma and smoking during pregnancy (45, 46), our findings indicate that these signals are already embedded in the shared, conserved methylation signature between amnion and nasal epithelium. This suggests that *in utero* programming effects persist within the post-natal nasal epithelium and may be tractable longitudinally, as compelling targets for early asthma risk stratification. It is important to acknowledge the observational nature of this study precludes establishing causality between methylation patterns in the nasal and amnion tissue, *in utero* exposures and epithelial dysfunction. Furthermore, we acknowledge that our approach pre-selects for the conserved methylome, and therefore the extent to which *in utero* exposures differentially influence these tissues is unclear, and therefore we do not provide an estimate for the overall level of concordance for exposure related effects. The latter reflects another limitation of this study due to the limited sample size, and the incomplete sampling of the full methylome when using target capture assays.

In conclusion, our study advances the field by providing cogent evidence for using amniotic tissue to provide insight regarding developmental programming of the respiratory epithelium. As well as identifying potential mechanisms for intrinsic vulnerability, a similar strategy could be used to assess prenatal interventions to enhance respiratory epithelial innate immune function. These approaches circumvent the challenges of obtaining airway epithelial cells from newborns, providing opportunities for early risk stratification that may eventually contribute to interventions for childhood wheezing and asthma.

## Supplementary Material

Supplement 1 **Figure E1. Analysis Workflow.** This study was conducted in three main phases: Sample processing and sequencing (green), Bioinformatics analysis (blue), and Statistical analysis (pink). The statistical analysis phase was further divided into four key steps: Step 1 – Dimensional reduction using Principal Component Analysis (PCA); Step 2 – Differential methylation analysis at the regional level (differentially methylated regions, DMRs) associated with tissue differences; Step 3 – Differential methylation analysis at the CpG site level (differentially methylated positions, DMPs) associated with tissue differences; Step 4 – Identification of a conserved DMP signature, which was then used to assess associations with gestational exposures (maternal asthma and maternal smoking). MD: Mean Difference; FDR: False Discovery Rate; FC: Fold Change.
**Figure E2. Conserved methylation landscape across tissues.** (A) Histogram counts of CpGs annotated to genomic features for the conserved methylome. (B) Significantly enriched biological processes within the conserved methylome for CpGs annotate to nearest transcriptional start sites. Point size represents count of number of genes relative to total genes within the pathway (C) Raw sequencing coverage across the SFTPA1 genet coding region (top panel) and the SCGB3A1 coding region. Ticks in red are methylated reads and blue represents unmethylated reads. Height of the grey silhouettes represents sequencing coverage.
**Figure E3. Pathway enrichment of maternal asthma history.** (A) Significantly enriched biological processes within the conserved methylome for CpGs associated with maternal asthma. Point size represents count of number of genes relative to total genes within the pathway. (B) Gene Ontology (GO) enrichment was calculated using genes near CpG sites from the maternal asthma signature, followed by a similarity matrix analysis. The similarity heatmap displays GO terms associated with Biological Process (BP), with colour intensity representing the significance of enrichment.
**Figure E4. Pathway enrichment of maternal smoking exposure.** (A) Significantly enriched biological processes within the conserved methylome for CpGs associated with maternal smoking. Point size represents count of number of genes relative to total genes within the pathway. (B) Gene Ontology (GO) enrichment was calculated using genes near CpG sites from the maternal smoking signature, followed by a similarity matrix analysis. The similarity heatmap displays GO terms associated with Biological Process (BP), with colour intensity representing the significance of enrichment.

Supplement 2 **Table E1. Tissue-Specific Differentially Methylated Regions (DMRs) and Positions (DMPs).** Results of differential methylation analysis comparing amniotic and nasal epithelium.

Supplement 3 **Table E2. Conserved CpG Methylation Across Lung-Specific Genes.** List of 1,493,976 CpG loci showing conserved methylation patterns across tissues, with genomic feature annotations.

Supplement 4 **Table E3. CpG Sites Associated with Maternal Asthma.** Binary logistic regression analysis, adjusted for multiple comparisons (FDR ≤ 0.05), identified significant associations at CpG sites with maternal asthma (MA) and smoking exposure (SE).

## Figures and Tables

**Table 1.** Demographic characteristics of the Airway Epithelium Respiratory Illnesses and Allergy (AERIAL) study.

Variables	Categories

**Gender**	**Female** 50 % (n=42) **Male** 50 % (n=42)
**Ethnicity**	**Mother** Caucasian (51.2 %) Non-caucasian (48.8 %) **Father** Caucasian (44.1 %) Non-caucasian (55.9 %)
**Smoking exposure**	**Yes** 4.76 % (n=4) **No** 95.54 % (n=80)
**Maternal asthma**	**Maternal asthma history** 11.91 % (n=10) **No maternal asthma history** 88.09 % (n=74)
**Gestational age** (mean ± SD)	39.12 ± 1.28 weeks
**Delivery Mode**	**Vaginal delivery** 47.62% (n=40) **Caesarean delivery** 47.62% (n=40) ***NA*** 4.76% (n=4)

***SD**: standard deviation; **NA**:* data not available

**Table 2.** Sequencing statistics for 84 matched samples from neonatal nasal and maternal amnion.

	Nasal samples	Amnion samples

**M seq** (in million)	168.67 ± 33.50	183 ± 28.90
**% GC**	27 ± 0.01	27% ± 0.01
**% Duplication**	15% ± 0.03	18% ± 0.03
**Insert Size**	225.35 bp ± 21.02	212.95 bp ± 9.98
**% Target Bases 10X**	99% ± 0.00	99% ± 0.00
**% Target Bases 30X**	93% ± 5.73	93% ± 8.55

bp: base pair

Values represent the mean ± standard deviation.

**Table 3.** Top 10 Most Differentially Methylated Positions (DMPs) in Maternal Asthma History.

Position	Symbol*	Log_2_FC	Adj. p-value

chr16:89629809	*DPEP1*	2.98	0.049936
chr17:74802258	*TMEM104*	2.36	0.02597409
chr10:13502863	*BEND7*	2.34	0.02597409
chr15:60787177	*RORA*	2.28	0.03648546
chr15:60787176	*RORA*	2.28	0.02597409
chr10:3332958		−1.45	0.00505511
chr6:169655096	*WDR27*	−1.46	0.04672635
chr18:45723869	SLC14A1; LOC105372092; LOC105372093	−2.22	0.01668038
chr18:45723868	SLC14A1; LOC105372092; LOC105372093	−2.27	0.02282406
chrX:70149858	*IGBP1*	−2.61	0.01668038

* CpG sites without a gene symbol may be located in regions with incomplete or less characterized gene annotations.

**Table 4.** Top 10 Most Differentially Methylated Positions (DMPs) in Maternal Smoking Exposure.

Position	Symbol*	Log_2_FC	Adj. p-value

chr8:143251263	*ZFP41*	4.99	0.02767552
chr6:15500540	*JARID2*	4.77	0.019276332
chr11:17779027	*KCNC1*; LOC107984317	4.74	0.015961482
chr8:143229735		4.73	0.012421924
chr14:74362734		4.66	5.60224E-07
chr7:158796784	*ESYT2*	−4.14	0.0415906
chr6:65566339	*EYS*	−4.20	0.008385907
chr12:31887708	LINC02422	−4.20	0.028869893
chr12:31887707	LINC02422	−4.47	0.011409155
chr10:129774152		−4.84	0.002327469

* CpG sites without a gene symbol may be located in regions with incomplete or less characterized gene annotations.
