# Supplementary material for: Programming of the respiratory epithelium in utero - insight from the amniotic epithelial methylome
Source: medRxiv. 2025 Oct 3:2025.10.02.25337047. Preprint. [Version 1] doi: 10.1101/2025.10.02.25337047 (PMC12622113; doi:10.1101/2025.10.02.25337047)
Supplement: Supplement 1 — Figure E1. Analysis Workflow. This study was conducted in three main phases: Sample processing and sequencing (green), Bioinformatics analysis (blue), and Statistical analysis (pink). The statistical analysis phase was further divided into four key steps: Step 1 – Dimensional reduction using Principal Component Analysis (PCA); Step 2 – Differential methylation analysis at the regional level (differentially methylated regions, DMRs) associated with tissue differences; Step 3 – Differential methylation analysis at the CpG site level (differentially methylated positions, DMPs) associated with tissue differences; Step 4 – Identification of a conserved DMP signature, which was then used to assess associations with gestational exposures (maternal asthma and maternal smoking). MD: Mean Difference; FDR: False Discovery Rate; FC: Fold Change. Figure E2. Conserved methylation landscape across tissues. (A) Histogram counts of CpGs annotated to genomic features for the conserved methylome. (B) Significantly enriched biological processes within the conserved methylome for CpGs annotate to nearest transcriptional start sites. Point size represents count of number of genes relative to total genes within the pathway (C) Raw sequencing coverage across the SFTPA1 genet coding region (top panel) and the SCGB3A1 coding region. Ticks in red are methylated reads and blue represents unmethylated reads. Height of the grey silhouettes represents sequencing coverage. Figure E3. Pathway enrichment of maternal asthma history. (A) Significantly enriched biological processes within the conserved methylome for CpGs associated with maternal asthma. Point size represents count of number of genes relative to total genes within the pathway. (B) Gene Ontology (GO) enrichment was calculated using genes near CpG sites from the maternal asthma signature, followed by a similarity matrix analysis. The similarity heatmap displays GO terms associated with Biological Process (BP), with colour intensity repres [file media-1.pdf]

Programming of the respiratory epithelium *in utero* - insight from the amniotic epithelial methylome

Patricia Agudelo-Romero[1,2,3*†], Thomas Iosifidis[1,4,5,*], James Lim[1], Nina Kresoje[1], David G. Hancock[1,6,7], Guicheng Zhang[1,4], Abhinav Sharma[8], Talya Conradie[1], Yuliya V. Karpievitch[1,4,9], Desiree T Silva[7,10,11,12], Anthony Bosco[13,14,] Susan L. Prescott[7,10,15,16], Peter N. LeSouëf [1,17], Elizabeth Kicic-Starcevich[1], Anthony Kicic[1,5,18,19], David J. Martino[1,9,#], Stephen M. Stick[6,#] .

[*]co-first authors, [#]co-senior authors, [†]corresponding author

Affiliations:

1: Wal-yan Respiratory Research Centre, The Kids Research Institute Australia, Perth, 6009, Western Australia, Australia.

2: School of Molecular Sciences, The University of Western Australia, Perth, Western Australia 6009, Australia.

3: European Virus Bioinformatics Centre, Friedrich-Schiller-Universitat Jena, 07737 Jena, Germany.

4: School of Population Health, Curtin University, Perth, 6102, Western Australia, Australia.

5: Centre for Cell Therapy and Regenerative Medicine, School of Medicine and Pharmacology, The University of Western Australia, Perth, 6009, Western Australia, Australia.

6: Department of Respiratory and Sleep Medicine, Perth Children's Hospital Perth, Perth, 6009, Western Australia, Australia.

7: Medical School, The University of Western Australia, Perth, 6009, Western Australia, Australia.

8: DSI-NRF Centre of Excellence for Biomedical Tuberculosis Research, SAMRC Centre for Tuberculosis Research, Division of Molecular Biology and Human Genetics, Faculty of Medicine and Health Sciences, Stellenbosch University, Cape Town, 7700, South Africa.

9: School of Biomedical Sciences, The University of Western Australia, Perth, 6009, Western Australia, Australia.

10: The Kids Research Institute Australia, Perth, 6009, Western Australia, Australia.

11: School of Medical and Health Sciences, Edith Cowan University, Perth, 6027, Western Australia, Australia.

12: Department of Paediatrics and Neonatology, Joondalup Health Campus, Perth, 6027, Western Australia, Australia.

13: Asthma and Airway Disease Research Center University of Arizona, Tucson, 85721, Arizona, United States.

14: Department of Immunobiology, The University of Arizona College of Medicine, Tucson, 85721, Arizona, United States.

15: Nova Institute for Health, Baltimore, 21231, Maryland, United States.

16: Department of Family and Community Medicine, University of Maryland, Baltimore, 21231, Maryland, United States.

17: School of Paediatrics and Child Health, University of Western Australia, Perth, 6027, Western Australia, Australia.

18: Occupation, Environment and Safety, School of Population Health, Curtin University, Perth, 6102, Western Australia, Australia.

19: Department of Respiratory and Sleep Medicine, Perth Children's Hospital, Perth, 6009, Western Australia, Australia.

***Co-first authors**

#Co-senior authors

†Correspondence:

To whom correspondence should be addressed: Dr Patricia Agudelo-Romero; Address: The Kids Research Institute Australia, Perth Children's Hospital, 15 Hospital Avenue, Nedlands, Western Australia, 6009; Email: patricia.agudeloromero@thekids.org.au

Supplementary Material

Figure E1. Analysis Workflow. This study was conducted in three main phases: Sample processing and sequencing (green), Bioinformatics analysis (blue), and Statistical analysis (pink). The statistical analysis phase was further divided into four key steps: Step 1 – Dimensional reduction using Principal Component Analysis (PCA); Step 2 – Differential methylation analysis at the regional level (differentially methylated regions, DMRs) associated with tissue differences; Step 3 – Differential methylation analysis at the CpG site level (differentially methylated positions, DMPs) associated with tissue differences; Step 4 – Identification of a conserved DMP signature, which was then used to assess associations with gestational exposures (maternal asthma and maternal smoking). MD: Mean Difference; FDR: False Discovery Rate; FC: Fold Change.

Figure E2. Conserved methylation landscape across tissues. (A) Histogram counts of CpGs annotated to genomic features for the conserved methylome. (B) Significantly enriched biological processes within the conserved methylome for CpGs annotate to nearest transcriptional start sites. Point size represents count of number of genes relative to total genes within the pathway (C) Raw sequencing coverage across the SFTPA1 genet coding region (top panel) and the SCGB3A1 coding region. Ticks in red are methylated reads and blue represents unmethylated reads. Height of the grey silhouettes represents sequencing coverage.

Figure E3. Pathway enrichment of maternal asthma history. (A) Significantly enriched biological processes within the conserved methylome for CpGs associated with maternal asthma. Point size represents count of number of genes relative to total genes within the pathway. (B) Gene Ontology (GO) enrichment was calculated using genes near CpG sites from the maternal asthma signature, followed by a similarity matrix analysis. The similarity heatmap displays GO terms

associated with Biological Process (BP), with colour intensity representing the significance of enrichment.

Figure E4. Pathway enrichment of maternal smoking exposure. (A) Significantly enriched biological processes within the conserved methylome for CpGs associated with maternal smoking. Point size represents count of number of genes relative to total genes within the pathway. (B) Gene Ontology (GO) enrichment was calculated using genes near CpG sites from the maternal smoking signature, followed by a similarity matrix analysis. The similarity heatmap displays GO terms associated with Biological Process (BP), with colour intensity representing the significance of enrichment.

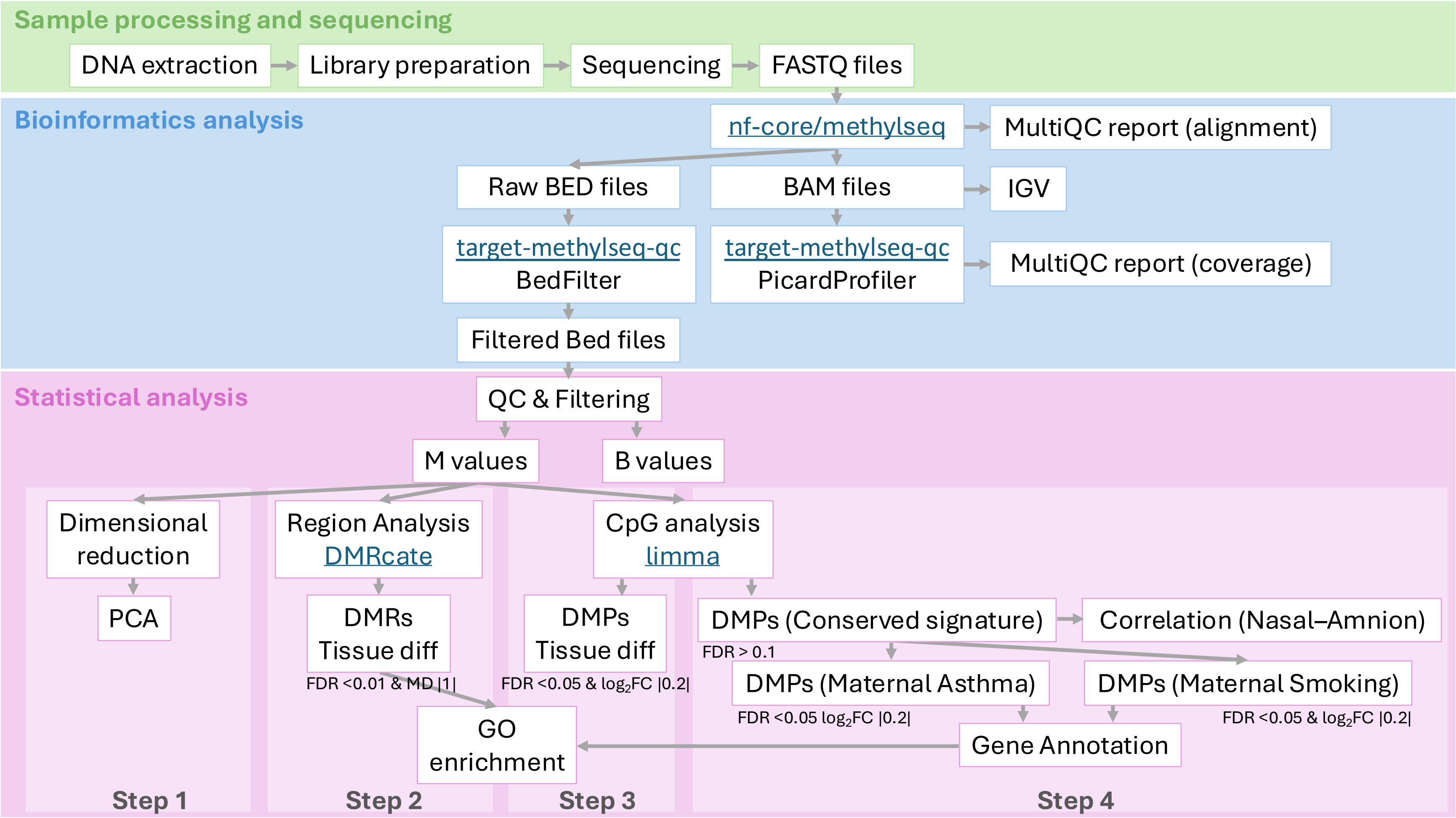

Figure E1. Analysis Workflow. This study was conducted in three main phases: Sample processing and sequencing (green), Bioinformatics analysis (blue), and Statistical analysis (pink). The statistical analysis phase was further divided into four key steps: Step 1 – Dimensional reduction using Principal Component Analysis (PCA); Step 2 – Differential methylation analysis at the regional level

(differentially methylated regions, DMRs) associated with tissue differences; Step 3 – Differential methylation analysis at the CpG site level (differentially methylated positions, DMPs) associated with tissue differences; Step 4 – Identification of a conserved DMP signature, which was then used to assess associations with gestational exposures (maternal asthma and maternal smoking). MD: Mean Difference; FDR: False Discovery Rate; FC: Fold Change.

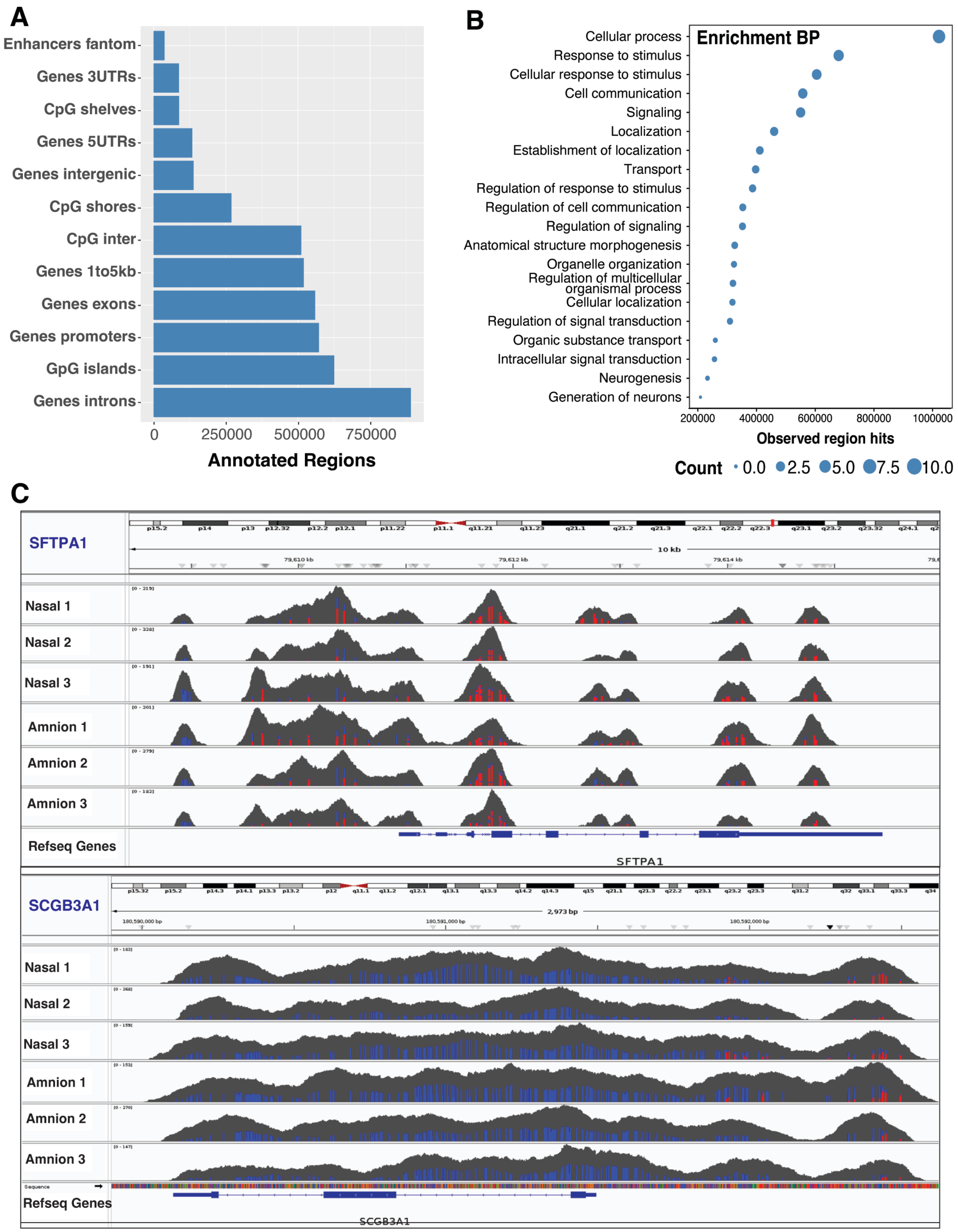
A
Enhancers fantom
Genes 3UTRs
CpG shelves
Genes 5UTRs
Genes intergenic
CpG shores
CpG inter
Genes 1to5kb
Genes exons
Genes promoters
GpG islands
Genes introns
0
250000
500000
750000
Annotated Regions
B
Enrichment BP
Cellular process
Response to stimulus
Cellular response to stimulus
Cell communication
Signaling
Localization
Establishment of localization
Transport
Regulation of response to stimulus
Regulation of cell communication
Regulation of signaling
Anatomical structure morphogenesis
Organelle organization
Regulation of multicellular organismal process
Cellular localization
Regulation of signal transduction
Organic substance transport
Intracellular signal transduction
Neurogenesis
Generation of neurons
200000
400000
600000
800000
1000000
Observed region hits
Count
0.0
2.5
5.0
7.5
10.0
C
SFTPA1
10 kb
Nasal 1
Nasal 2
Nasal 3
Amnion 1
Amnion 2
Amnion 3
Refseq Genes
SFTPA1
SCGB3A1
2,973 bp
Nasal 1
Nasal 2
Nasal 3
Amnion 1
Amnion 2
Amnion 3
Refseq Genes
SCGB3A1

Figure E2. Conserved methylation landscape across tissues. (A) Histogram counts of CpGs annotated to genomic features for the conserved methylome. (B) Significantly enriched biological processes within the conserved methylome for CpGs annotate to nearest transcriptional start sites. Point size represents count of number of genes relative to total genes within the pathway (C) Raw sequencing coverage across the SFTPA1 genet coding region (top panel) and the SCGB3A1 coding region. Ticks in red are methylated reads and blue represents unmethylated reads. Height of the grey silhouettes represents sequencing coverage.

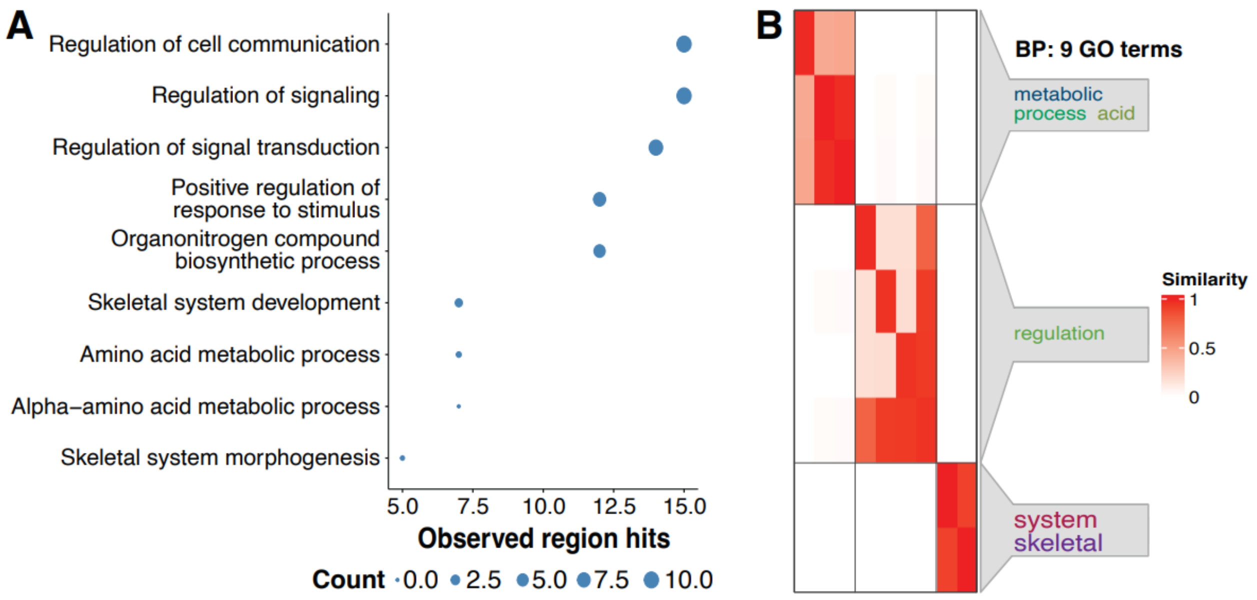

Figure E3. Pathway enrichment of maternal asthma history. (A) Significantly enriched biological processes within the conserved methylome for CpGs associated with maternal asthma. Point size represents count of number of genes relative to total genes within the pathway. (B) Gene Ontology (GO) enrichment was calculated using genes near CpG sites from the maternal asthma signature, followed by a similarity matrix analysis. The similarity heatmap displays GO terms associated with Biological Process (BP), with colour intensity representing the significance of enrichment.

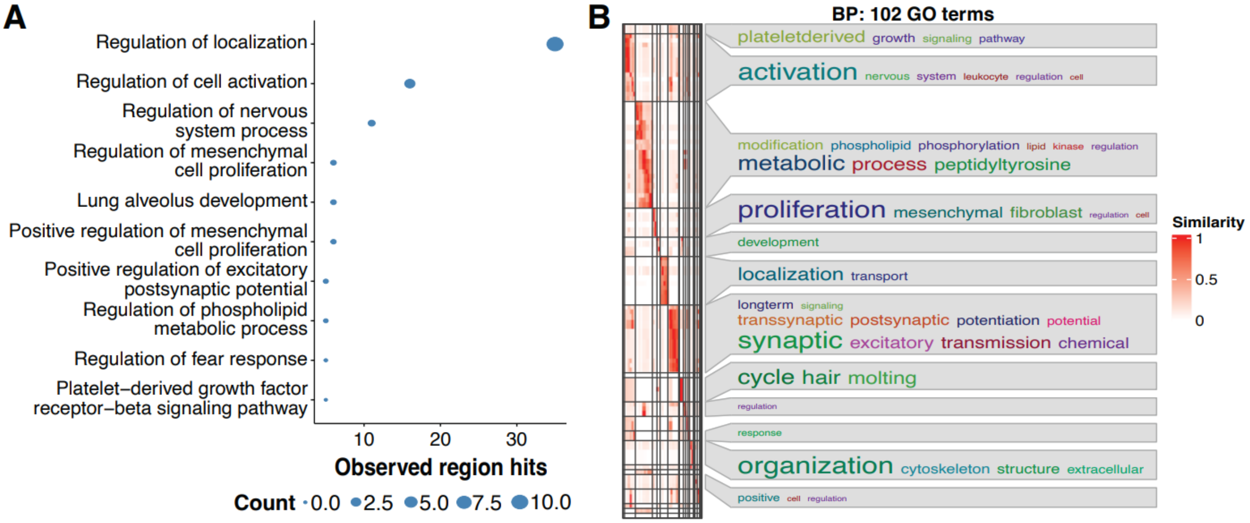

Figure E4. Pathway enrichment of maternal smoking exposure. (A) Significantly enriched biological processes within the conserved methylome for CpGs associated with maternal smoking. Point size represents count of number of genes relative to total genes within the pathway. (B) Gene Ontology (GO) enrichment was calculated using genes near CpG sites from the maternal smoking signature, followed by a similarity matrix analysis. The similarity heatmap displays GO terms associated with Biological Process (BP), with colour intensity representing the significance of enrichment.
